# The Therapeutic Potential and Mechanisms of Action of Quercetin in Relation to Lipopolysaccharide-Induced Sepsis In Vitro and In Vivo

**DOI:** 10.1371/journal.pone.0080744

**Published:** 2013-11-19

**Authors:** Yu-Cheng Chang, Ming-Han Tsai, Wayne Huey-Herng Sheu, Shu-Chen Hsieh, An-Na Chiang

**Affiliations:** 1 Institute of Biochemistry and Molecular Biology, National Yang-Ming University, Taipei, Taiwan; 2 Department of Internal Medicine, Taichung Veterans General Hospital, Taichung, Taiwan; 3 Institute of Food Science and Technology, National Taiwan University, Taipei, Taiwan; University of Cincinnati, United States of America

## Abstract

Sepsis caused by Gram-negative bacterial infection is characterized by extensive inflammatory cytokine production, which leads to multiple organ failure and a high lethality rate. Therefore, compounds that are able to alleviate profound inflammatory responses may have therapeutic potential in relation to sepsis. Quercetin, one of the flavonoids found widely in the human diet, has been reported to have many health benefits, but the mechanisms underlying its biological effects remain obscure. In the present study, our aim was to investigate the molecular mechanisms by which quercetin inhibits lipopolysaccharide (LPS)-induced pro-inflammatory cytokine production and to evaluate the capacity of quercetin to attenuate the mortality rate in a mice model of lethal sepsis. Our results show that quercetin significantly attenuates LPS-induced production of tumor necrosis factor-α (TNF-α) and interleukin-1β (IL-1β) in RAW264.7 macrophages. The LPS-stimulated phosphorylations of the inhibitors of κB kinase (IKKs), Akt, and c-Jun N-terminal kinase (JNK) are also inhibited by quercetin. Quercetin causes a significant reduction in the phosphorylation and degradation of inhibitor of κBα (IκBα) and in the nuclear level of nuclear factor-κB (NF-κB), the latter being associated with decreased NF-κB binding activity. Most importantly, acute administration of quercetin reduces the lethality rate and circulating levels of TNF-α and IL-1β in C57BL/6J mice with endotoxemia induced by LPS, whereas chronic dietary supplementation with quercetin shows no inhibitory effect on serum TNF-α and IL-1β levels. These findings provide clues that quercetin may be a promising agent for the prevention of systemic inflammatory diseases such as sepsis.

## Introduction

Sepsis, a life-threatening disease with a high mortality rate, is accompanied by systemic inflammation with excessive production of pro-inflammatory cytokines including tumor necrosis factor-α (TNF-α) and interleukin-1β (IL-1β) [1]. Endotoxin, the outer membrane component of Gram-negative bacteria, is a major pathogenic factor in sepsis [2]. Lipopolysaccharide (LPS) infusion/injection has been established for sepsis research because LPS induces systemic inflammation mimicking the initial clinical features of sepsis [3]. Significant advances have been made in the therapeutic strategies used to treat sepsis, but the mortality rate has not substantially improved [4]. Previous studies have reported that flavonoids are able to protect rats and mice from LPS-induced tissue damage and lethal septic shock [5,6]. It seems that flavonoids may have beneficial effects on the prevention of sepsis. Quercetin, one of the most widely distributed flavonoids in plants, is a major constituent in the human diet [7]. Quercetin has a broad range of biological functions associated with the modulation of oxidative stress and inflammatory response [8,9]. Recent studies have indicated that quercetin is able to reduce the release of TNF-α and IL-1β, thereby alleviating inflammatory responses [10,11]. Despite the fact that the anti-inflammatory function of quercetin is known, the role of quercetin in the prevention of mortality and systemic inflammation in animals with lethal sepsis still remains to be elucidated.

Stimulation of Toll-like receptor 4 (TLR4) by LPS activates downstream inhibitors of the κB kinase (IKKs), mitogen-activated protein kinase (MAPK), and Akt signaling pathways [12,13]. IKKs (comprising IKKα, IKKβ, and IKKγ) are the upstream kinases of the inhibitory κB proteins (IκBα, IκBβ, and IκBε). Phosphorylation of IκBα by IKKs is a key regulatory step that leads to the degradation of IκBα and the subsequent translocation of nuclear factor-κB (NF-κB) to the nucleus; NF-κB then binds to the NF-κB sites of specific gene promoters thereby activating the expression of inflammatory cytokines [14]. It has been reported that eupatilin, a member of the flavonoids, suppresses the TNF-α-induced phosphorylation of IKK and IκBα in human bronchial epithelial cells [15]. Furthermore, an extract of *Ginkgo biloba* that is enriched with quercetin has been reported to attenuate the activation of NF-κB pathway in macrophages [16]. These studies suggest that quercetin may prevent the activation of the LPS-induced NF-κB pathway. 

The MAPK family consists of extracellular signal-regulated kinases 1/2 (ERK1/2), c-Jun N-terminal kinase 1/2 (JNK1/2), and p38 [17]. A recent study has indicated that blockade of NF-κB and MAPK activation protects mice from tissue injury during sepsis and also reduces the production of pro-inflammatory cytokines [18]. In addition, suppression of Akt activation has been shown to reduce the LPS-induced inflammatory responses in human endothelial cells [19]. Nevertheless, even taking the above findings into account, little is known about the role of quercetin in the regulation of LPS-induced NF-κB, MAPK, and Akt activation in macrophages.

In the present study, our aim was to investigate whether quercetin inhibits LPS-induced pro-inflammatory cytokine production in parallel with the LPS-stimulated signaling pathways in macrophages. We also evaluated whether quercetin is able to inhibit cytokine release and thus attenuate the mortality rate in animals with lethal sepsis. Our results clearly show that quercetin is able to suppress the LPS-induced TNF-α and IL-1β production by modulating the NF-κB, JNK, and Akt signaling pathways, and alleviate lethality in mice with LPS injection. Our results provide evidence that quercetin is able to effectively protect mice from sepsis-related systemic inflammation and mortality.

## Materials and Methods

### Ethics statement

All animal experiments were approved by the Animal Care and Ethics Committee of National Yang-Ming University and followed the guidelines by the U.S. National Institutes of Health Guide for the Care and Use of Laboratory Animals.

### Reagents and antibodies

Quercetin dihydrate and lipopolysaccharide (LPS, *Escherichia coli* 0111:B4) were obtained from Sigma-Aldrich (St. Louis, MO, USA). Anti-p50, α-tubulin, and B23 antibodies were purchased from Abcam (Cambridge, UK). Antibodies against phospho-IKK, phospho-Akt, phospho-JNK1/2, phospho-ERK1/2, phospho-p38, phospho-IκBα, total IKK, total Akt, total JNK1/2, total ERK1/2, and total p38 were obtained from Cell Signaling Technology (Beverly, MA, USA). Anti-p65 antibody was purchased from Upstate Biotechnology (Lake Placid, NY, USA). Antibody against IκBα was obtained from Santa Cruz Biotechnology (Santa Cruz, CA, USA).

### Cell cultures and treatments

Murine macrophage RAW264.7 cells were originally obtained from the American Type Culture Collection (Manassas, VA, USA) and maintained in Dulbecco’s modified Eagle’s medium (DMEM; Hyclone Laboratories, Logan, UT, USA) supplemented with 10% fetal bovine serum (FBS; PAA Laboratories GmbH Linz, Austria), 100 units/ml penicillin, and 100 μg/ml streptomycin. Cell cultures were performed in a humidified atmosphere at 37°C with 5% CO_2_. Quercetin dihydrate was dissolved in dimethyl sulfoxide and diluted using medium containing 1% FBS to achieve the final concentrations. RAW264.7 macrophages were incubated with different concentrations of quercetin for 1 h followed by treatment with 100 ng/ml LPS.

### Enzyme-linked immunosorbent assay (ELISA)

The levels of TNF-α and IL-1β in serum and in culture medium were determined using ELISA kits (R&D systems, Minneapolis, MN, USA) according to the manufacturer’s instructions.

### RNA extraction and quantitative real-time PCR

Total cellular RNA was extracted using the TRI Reagent (Sigma-Aldrich) following the manufacturer’s protocol. First-strand cDNA was synthesized from 2 μg of total RNA using moloney murine leukemia virus reverse transcriptase (Invitrogen, Carlsbad, CA, USA). Quantitative real-time PCR was performed using SYBR Green PCR Master Mix (Finnzymes, Espoo, Finland) on a Roche LightCycler system (Roche Diagnostics, Mannheim, Germany). The primer sequences used for PCR amplification were as follows: TNF-α (forward 5′-CCTGTGAGGAGGACGAACAT-3′ and reverse 5′-GAGGAAGGCCTAAGGTCCAC-3′), IL-1β (forward 5′-GCCTCGTGCTGTCGGACCCA-3′ and reverse 5′-TGAGGCCCAAGGCCACAGGT-3′), and glyceraldehyde 3-phosphate dehydrogenase (GAPDH, forward 5′-GTATGACTCCACTCACGGCAAA-3′ and reverse 5′-GGTCTCGCTCCTGGAAGATG-3′). The levels of *TNF-α* and *IL-1β* gene expression were normalized using GAPDH as the internal control.

### Western blot analysis

Total cell extracts were prepared by resuspending the cells in lysis buffer [20 mM Tris-HCl, pH 7.5, 150 mM NaCl, 1 mM EDTA, 1 mM EGTA, 1% Triton X-100, 50 mM dithiothreitol, complete protease inhibitor cocktail (Roche Diagnostics) and phosphatase inhibitor cocktail I and II (Sigma-Aldrich)]. Total cell lysates were centrifuged and the supernatants were collected. The protein concentration was determined using Bradford reagent (Bio-Rad, Hercules, CA) with bovine serum albumin (BSA; Sigma-Aldrich) as the standard. Equal amounts of protein were separated by SDS-PAGE and transferred to nitrocellulose membranes (Pall, Glen Cove, NY, USA). The immunoblots were then probed with the specific antibody overnight at 4°C followed by incubating with goat anti-rabbit or mouse IgG horseradish peroxidase conjugated antibodies (Sigma-Aldrich). The protein bands were detected using the enhanced chemiluminescence method (ECL, PerkinElmer, Boston, MA, USA). The blots were then stripped for further probing with α-tubulin or B23 antibody as the internal control for total cellular protein or nuclear protein, respectively. The relative intensity of the protein bands was quantified using Image Quant software (Molecular Dynamics, Sunnyvale, CA, USA).

### Nuclear extract preparation

Cells were lysed by incubating with hypotonic lysis buffer [20 mM HEPES, pH 7.4, 10 mM KCl, 1 mM MgCl_2_, 0.5% NP-40, 0.5 mM dithiothreitol, and complete protease inhibitor cocktail (Roche Diagnostics)] on ice for 30 min. The nuclei were then pelleted by centrifugation and resuspended in high salt nuclear extraction buffer [20 mM HEPES, pH 7.4, 10 mM KCl, 1 mM MgCl_2_, 400 mM NaCl, 0.5 mM dithiothreitol, and complete protease inhibitor cocktail (Roche Diagnostics)] on ice for 30 min. The nuclear proteins were harvested after removal of the nuclear debris by centrifugation.

### Electrophoretic Mobility Shift Assay (EMSA)

Nuclear extracts (3 μg) were incubated with ^32^P-labeled double-stranded DNA fragment corresponding to NF-κB (sense strand, 5′-AGTTGAGGGGACTTTCCCAGGC-3′) in the presence or absence of unlabeled double-stranded DNA fragment corresponding to NF-κB for 10 min at room temperature. For the supershift assays, NF-κB p65 or p50 antibodies were incubated with nuclear extracts for 24 h before addition of the labeled oligonucleotide. The transcription factor-bound oligonucleotides were then separated on 6% non-denaturing polyacrylamide gels. The gel was dried and analyzed using a PhosphorImager (Molecular Dynamics).

### Animals

Male C57BL/6J mice (aged 7- to 8-week-old; Jackson Laboratories, Bar Harbor, ME, USA) were housed 2~3 animals per cage under temperature 22°C and 12 hr light/dark cycle with free access to food and water. Endotoxemia was induced by intraperitoneal injection of 10 mg/kg LPS as previously described [20]. To observe the effect of chronic quercetin administration, the mice (n=8 for each group) had quercetin, which had been prepared in 1% polyethylene glycol (Sigma-Aldrich) solution at a dose of 10 and 100 mg/kg, intragastrically administered for 10 consecutive weeks. Mice in the control group were treated with an equal amount of vehicle. After 10 weeks of quercetin treatment, the mice were intraperitoneally injected with 10 mg/kg LPS and retro-orbital blood was collected at 6 h after LPS challenge. Isoflurane was used as the inhalation anesthetic agent for blood collection. To observe the effects of acute quercetin administration, mice were intraperitoneally injected with a single dose of quercetin (0, 1, 10, 50, and 100 mg/kg) followed 1 h later by LPS stimulation. Retro-orbital blood was collected under anesthesia by inhalation of isoflurane at different times (one hour for TNF-α assay and eight hours for IL-1β assay) after LPS administration and allowed to clot at room temperature before centrifugation. Serum samples were assayed immediately or were stored at –30°C before analysis. Mortality was recorded for up to 4 weeks after LPS injection. All mice were carefully monitored and were humanely sacrificed under anaesthesia with 95% CO_2_/5% O_2_ when signs of illness were detected. 

### Statistical analysis

The results are presented as mean ± SD of at least three independent experiments. Statistical analysis was performed using Student’s *t* test or one-way ANOVA with the Tukey’s method as a *post hoc* test. Differences were considered statistically significant when *p*<0.05.

## Results

### Quercetin inhibits TNF-α and IL-1β secretion in macrophage cultures

**Figure 1:**
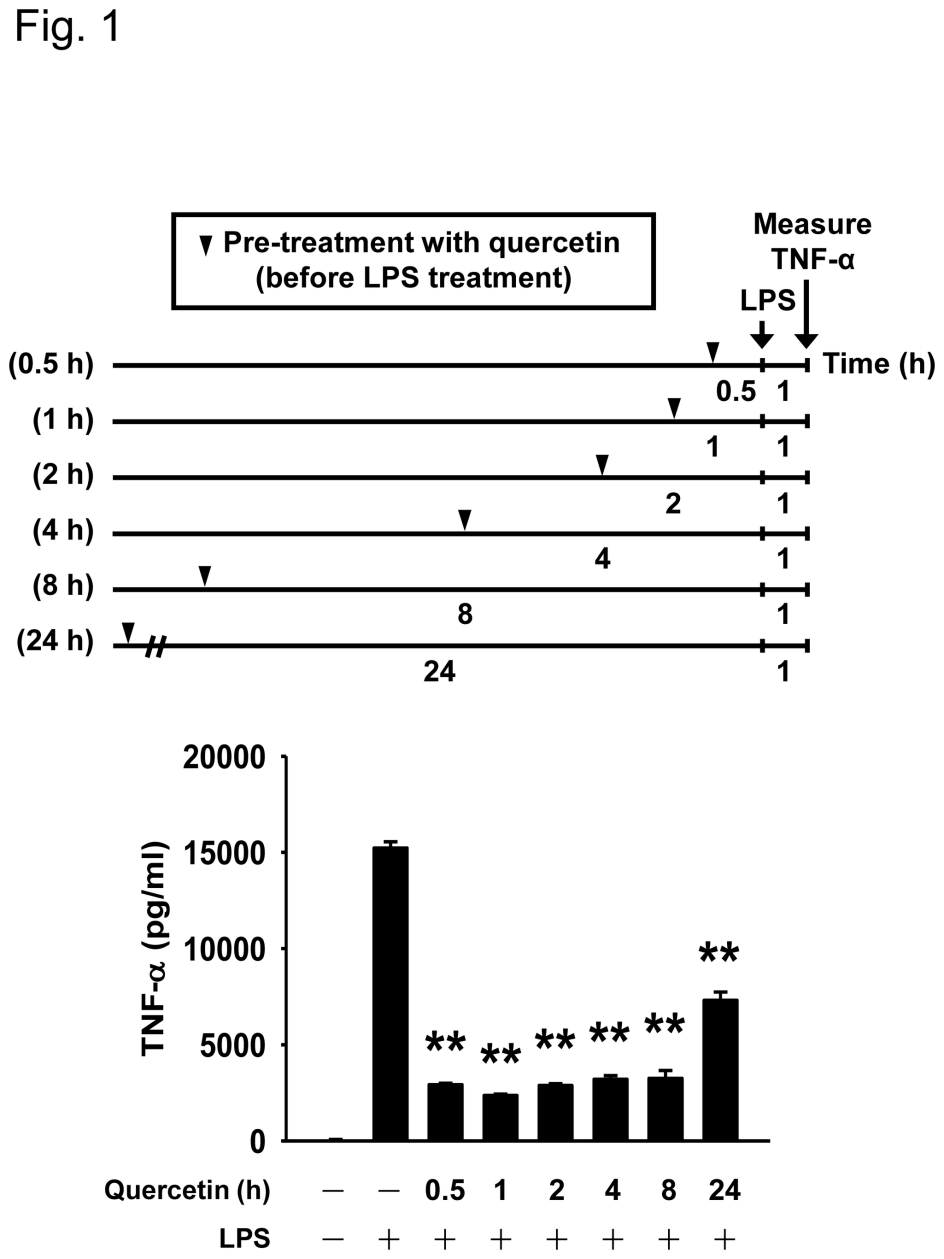
Time-dependent effect of quercetin on TNF-α secretion in RAW264.7 macrophages. Cells were pretreated with 30 μM quercetin for indicated times and then stimulated with 100 ng/ml LPS for 1 h. The level of TNF-α secreted from macrophages was measured by ELISA. Bars are mean ± SD (n = 3). **p<0.01 versus LPS alone group.

We first evaluated the effect of quercetin on TNF-α secretion by macrophages. RAW264.7 cells were pretreated with quercetin for different times followed by LPS treatment. The secretion of TNF-α was remarkably increased in the LPS-challenged group (15226 ± 329 pg/ml), while macrophages with quercetin pretreatment at various times before LPS stimulation (0.5, 1, 2, 4, 8, and 24 h) showed a significant inhibition of TNF-α secretion (2916 ± 71 pg/ml, 2356 ± 69 pg/ml, 2877 ± 94 pg/ml, 3201 ± 188 pg/ml, 3251 ± 412 pg/ml, and 7299 ± 437 pg/ml, respectively) (Figure 1). This observation indicates that macrophages pretreated with quercetin are able to exert anti-inflammatory actions.

**Figure 2:**
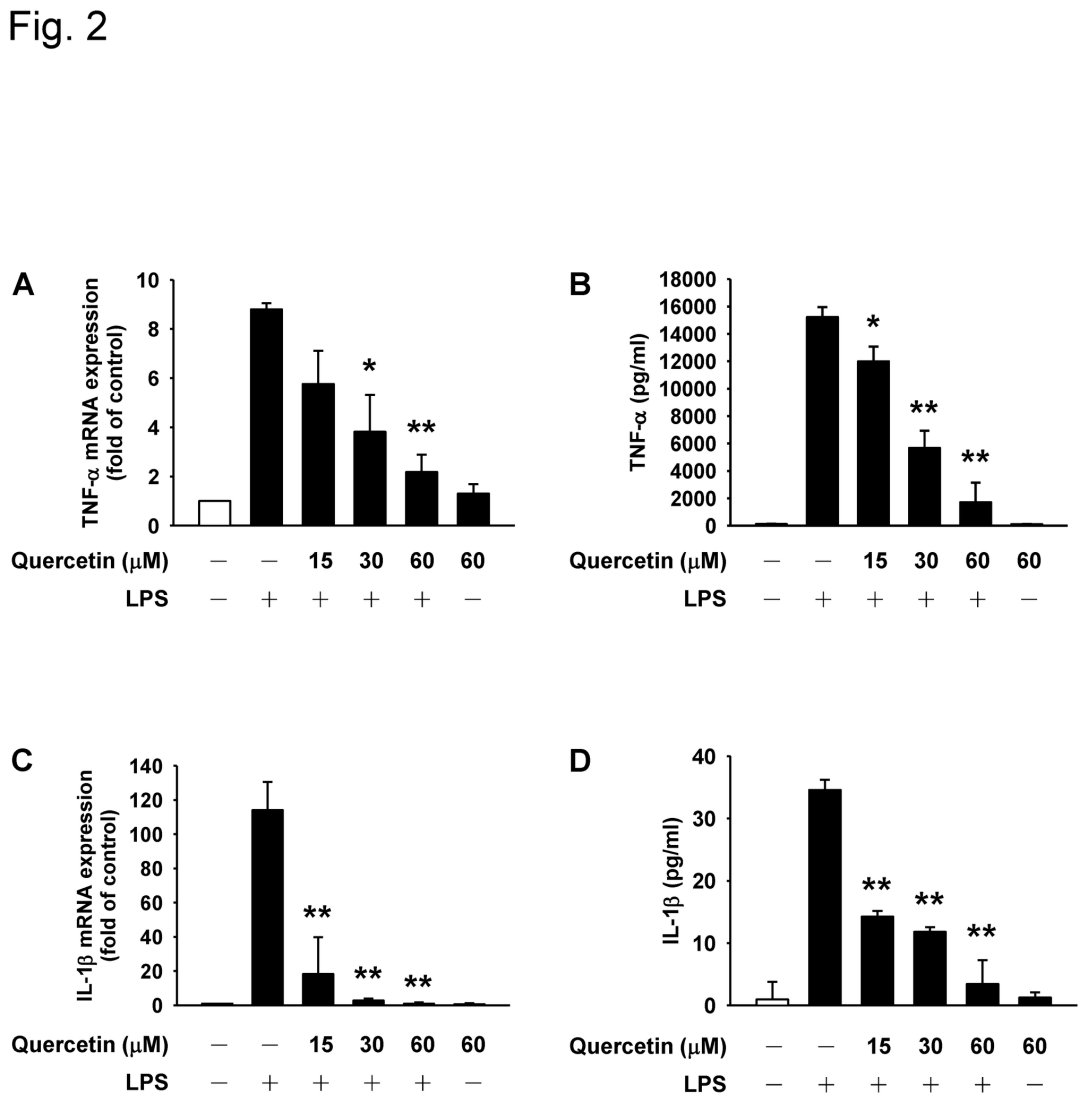
The role of quercetin in TNF-α and IL-1β expression in RAW264.7 macrophages. Cells were preincubated with different concentrations of quercetin as indicated for 1 h and then stimulated with 100 ng/ml LPS for another 1 h. The mRNA levels of TNF-α (**A**) and IL-1β (**C**) were analyzed by quantitative real-time PCR. The levels of TNF-α (**B**) and IL-1β (**D**) secreted from macrophages were measured by ELISA. Data are presented as the mean ± SD (n = 3). *p<0.05, **p<0.01 versus LPS alone group.

To assess the short-term effects of quercetin on LPS-induced production of TNF-α and IL-1β, RAW264.7 macrophages were treated with different concentrations of quercetin (15, 30, and 60 μM) for 1 h followed by LPS stimulation. Quercetin was found to dose-dependently suppress LPS-induced *TNF-α* and *IL-1β* gene expression (Figure 2A and 2C). We also examined whether the secretion of TNF-α and IL-1β levels were decreased by quercetin. In line with the effect of quercetin on *TNF-α* and *IL-1β* gene expression, pretreatment of cells with quercetin inhibited LPS-induced TNF-α and IL-1β secretion in a concentration-dependent manner (Figure 2B and 2D).

### Quercetin attenuates LPS-induced phosphorylation of IKK, Akt, and JNK

**Figure 3:**
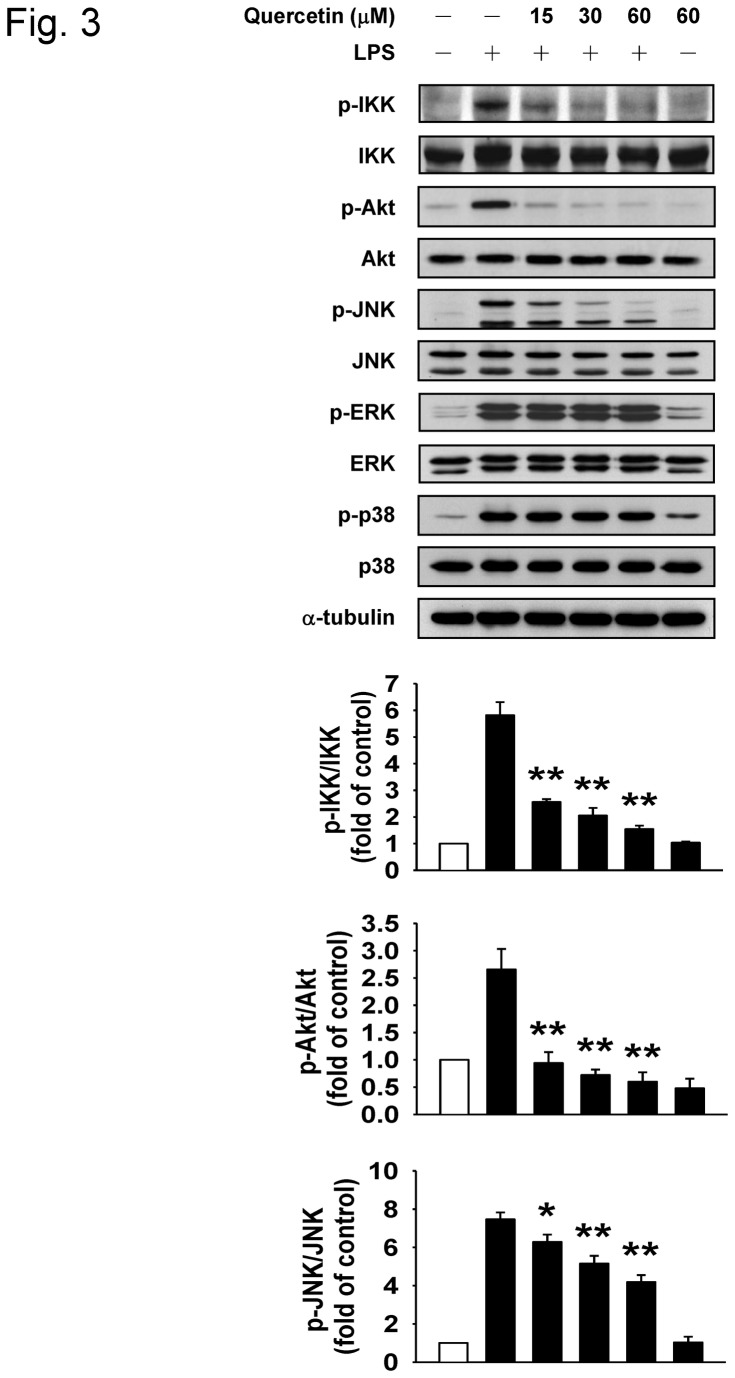
Effect of quercetin on the phosphorylation of IKK, Akt, and the MAPKs in LPS-stimulated RAW264.7 macrophages. Cells were pretreated with the indicated concentrations of quercetin for 1 h and then incubated with 100 ng/ml LPS for 30 min. Phosphorylated IKK (p-IKK), phosphorylated Akt (p-Akt), phosphorylated JNK (p-JNK), phosphorylated ERK (p-ERK), and phosphorylated p38 (p-p38) were detected by Western blot analysis. The intensity of the protein band was normalized against total IKK, Akt, JNK, ERK, and p38, respectively. The normalized levels of phosphorylated protein from control group were set as 1. The results represent mean ± SD (n = 3). *p<0.05, **p<0.01 versus LPS alone group.

To investigate the possible mechanism by which quercetin suppresses LPS-induced TNF-α and IL-1β expression, we determined the influence of quercetin on the LPS-induced activation of IKK, Akt, JNK, ERK, and p38. As shown in Figure 3, quercetin dose-dependently inhibited the phosphorylation of IKK, Akt, and JNK, whereas the phosphorylation levels of ERK and p38 were not affected by quercetin treatment. These findings suggest that quercetin exerts its anti-inflammatory effects via the inhibition of IKK, Akt, and JNK phosphorylations.

### Quercetin inhibits NF-κB activation in the LPS-stimulated macrophages

**Figure 4:**
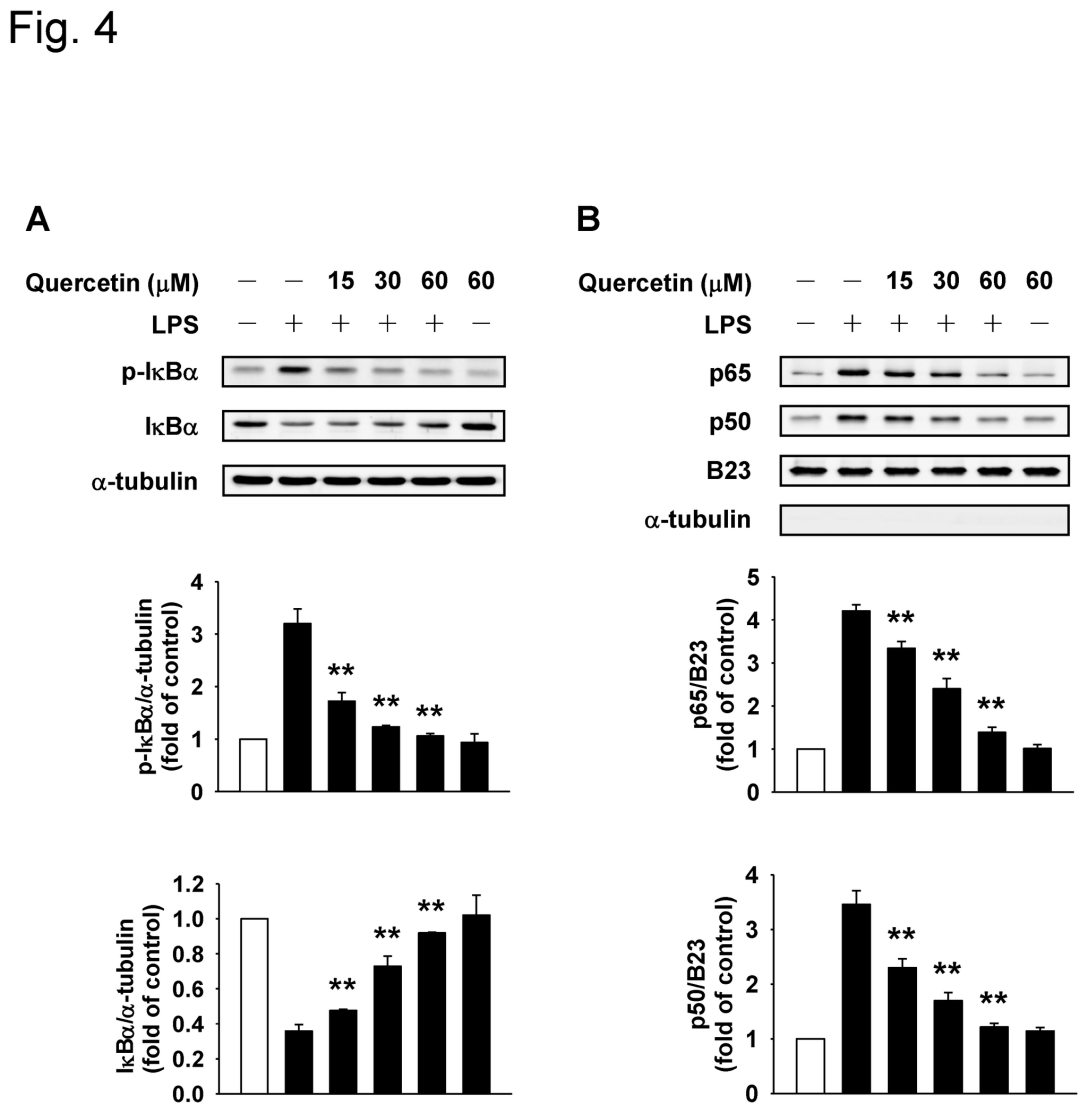
Quercetin inhibits LPS-induced IκBα phosphorylation, IκBα degradation, nuclear p65 expression and p50 expression in macrophages. Cells were pretreated with indicated concentrations of quercetin for 1 h before stimulation with 100 ng/ml LPS for 30 min. The levels of total and phosphorylated IκBα in the cell lysates (**A**) and of nuclear NF-κB, p65 and p50 (**B**) were determined by Western blot analysis. B23 was used as nuclear loading control, and α-tubulin was used to exclude cytosolic contamination. The normalized levels of protein from the control group are given the value of 1. Bars are mean ± SD (n = 3-4). **p<0.01 versus LPS alone group.

**Figure 5:**
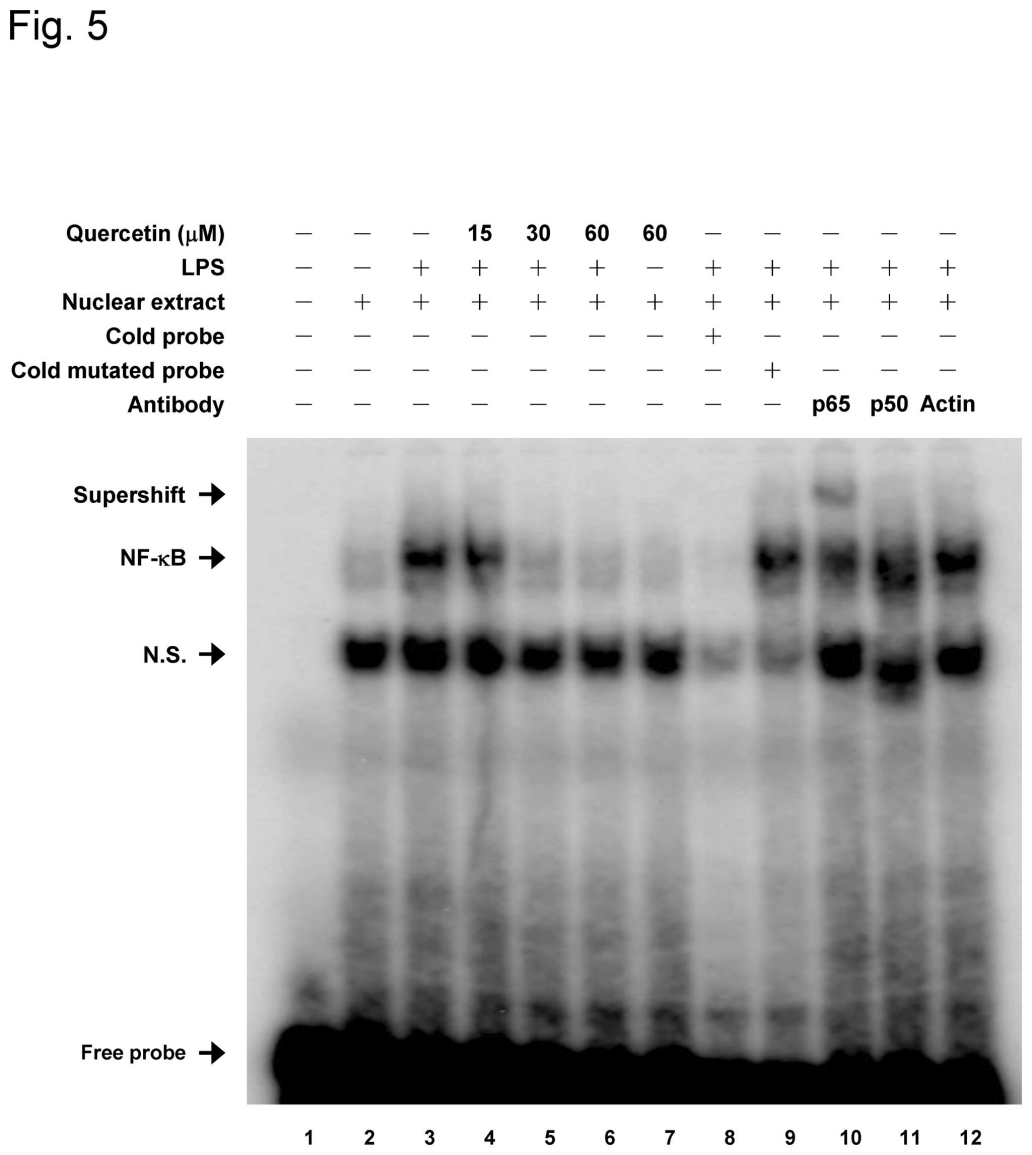
Quercetin attenuates LPS-stimulated NF-κB DNA binding activity. Cells were pretreated with the indicated concentrations of quercetin for 1 h and then incubated with 100 ng/ml LPS for 30 min. Nuclear extracts were harvested and analyzed by EMSA. DNA binding specificity was determined by addition of unlabeled NF-κB probe (cold probe), p65 or p50 antibodies to the nuclear extracts. The result shown is representative of three independent experiments.

It is well known that the IKKs are upstream kinases of IκB in the NF-κB signaling pathway [14] and therefore we next examined the effects of quercetin on LPS-induced NF-κB activation pathway in macrophages. As shown in Figure 4A, phosphorylation of IκBα was significantly induced in response to LPS stimulation, while pretreatment of cells with quercetin attenuated IκBα phosphorylation in a dose-dependent manner. Subsequently, degradation of IκBα was reversed by quercetin. We next examined the effect of quercetin on the nuclear levels of p65 and p50 in LPS-stimulated macrophages (Figure 4B). Nuclear p65 and p50 levels were significantly induced by LPS; nevertheless, quercetin dose-dependently reduced the LPS-induced levels of nuclear p65 and p50. We next investigated the effect of quercetin on NF-κB transcriptional activity. As shown in Figure 5, LPS-induced NF-κB DNA binding activity was dose-dependently suppressed by quercetin. The binding specificity was confirmed by competition with cold probe or cold mutated probe. A “supershift” band could be detected in the presence of p65 specific antibody, though the band shift was not clear in the presence of p50 specific antibody. Taken together, these results suggest that the inhibition of cytokine expression by quercetin is responsible for its suppression effect of NF-κB signaling pathway in macrophages.

### Quercetin prevents LPS-induced septic death and inhibits TNF-α and IL-1β release in mice

**Figure 6:**
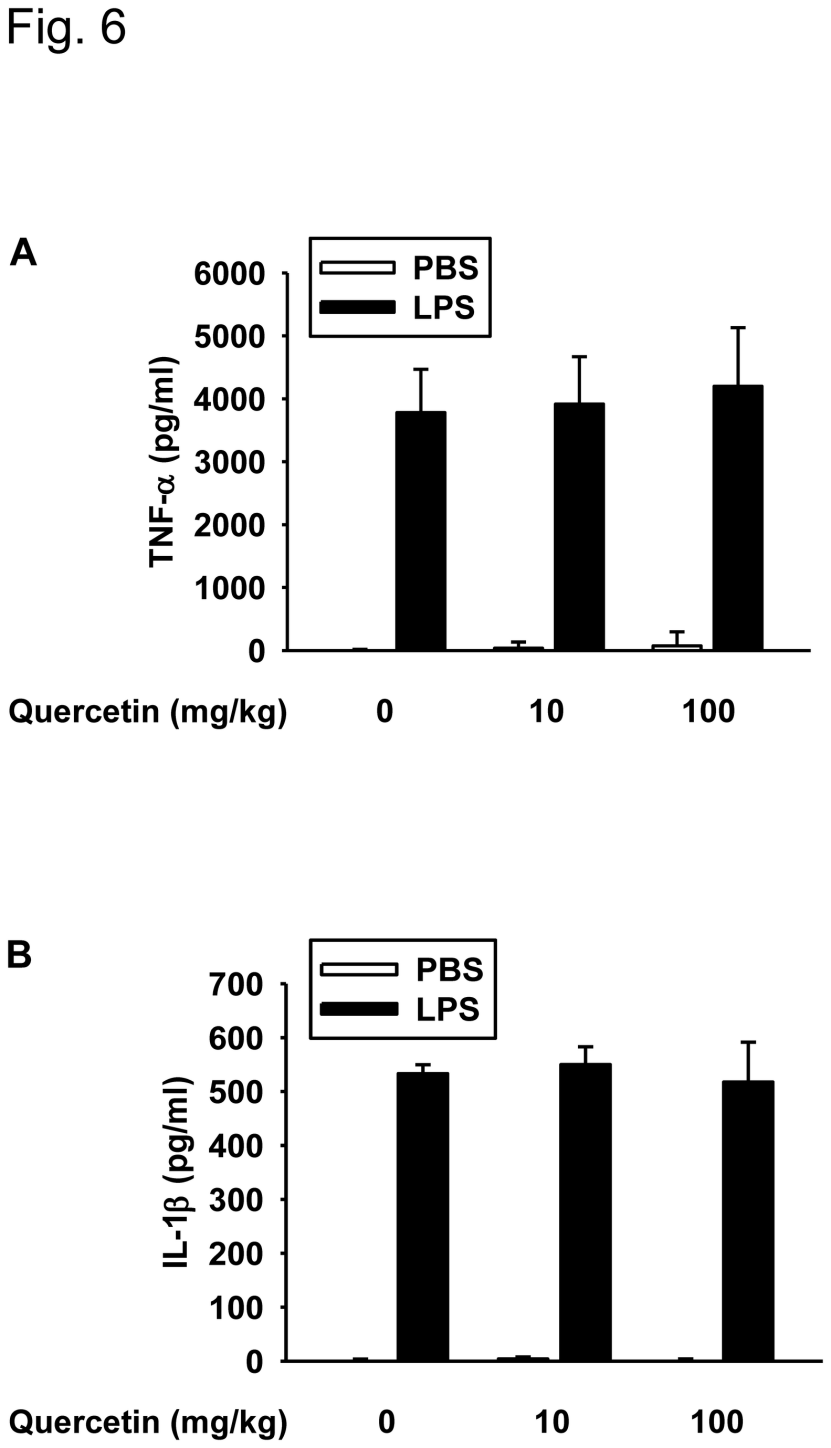
Effect of chronic quercetin administration on the secretion of pro-inflammatory cytokines in LPS-challenged C57BL/6J mice. Mice were daily given quercetin at a single dose of 10 and 100 mg/kg by gavage for 10 weeks. After 10 weeks of treatment, the mice were intraperitoneally injected with 10 mg/kg LPS. The serum concentrations of TNF-α (**A**) or IL-1β (**B**) at 6 h after the induction with LPS were measured by ELISA. Bars are mean ± SD (n = 8).

**Figure 7:**
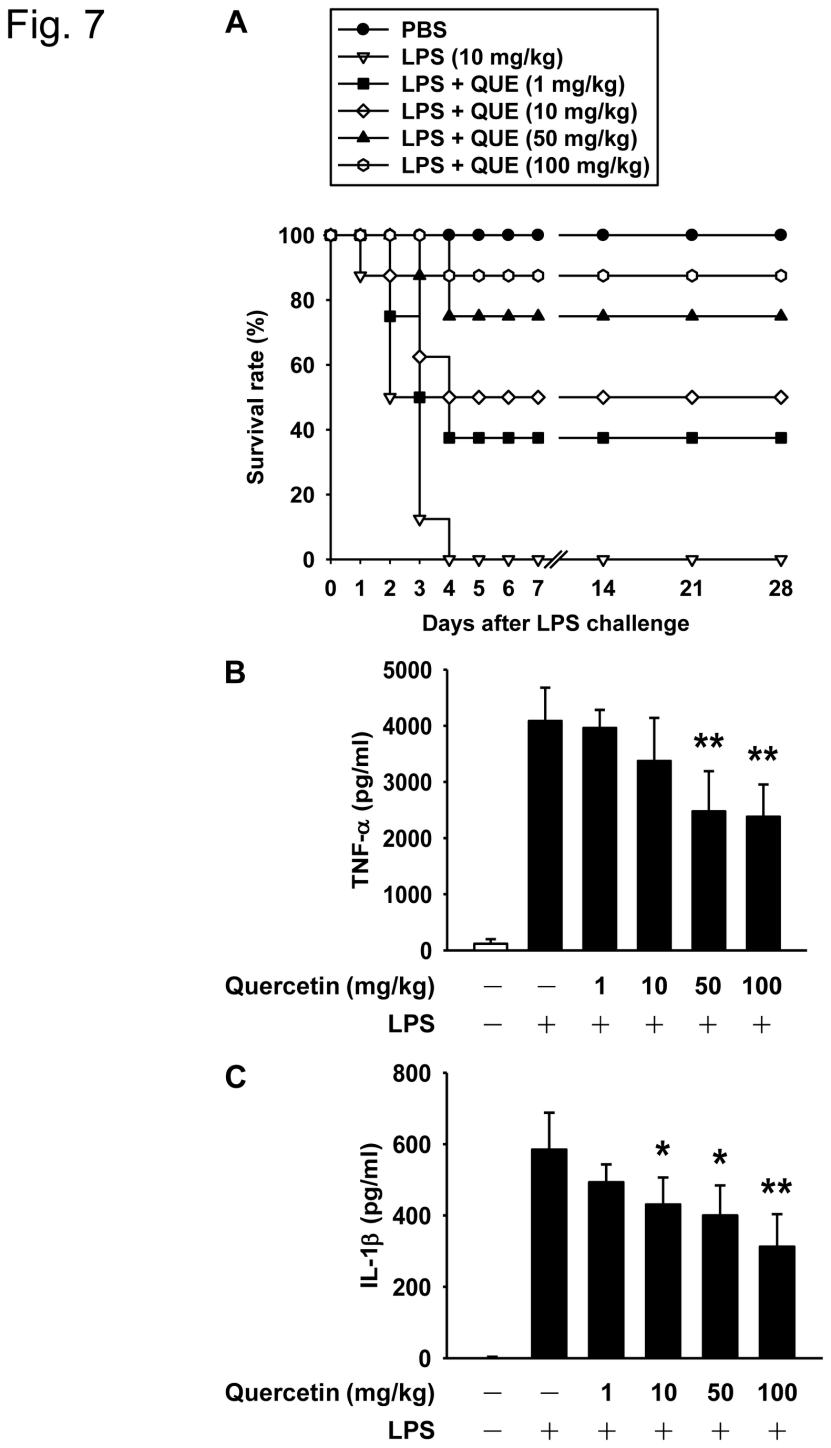
Acute administration of quercetin protects against LPS-induced mortality and pro-inflammatory cytokine secretion in C57BL/6J mice. **A**. Mice were intraperitoneally injected with a single dose of quercetin as indicated for 1 h followed by 10 mg/kg LPS stimulation and monitored for survival. The serum concentrations of TNF-α at 1 h (**B**) or IL-1β at 8 h (**C**) after the induction of LPS were measured by ELISA. Values are mean ± SD (n = 8). *p<0.05, **p<0.01 versus LPS alone group.

We assessed the chronic and acute administration effects of quercetin using a mice sepsis model. Although serum levels of TNF-α and IL-1β were remarkably induced in mice challenged by an intraperitoneal injection of 10 mg/kg LPS, chronic administration of 10 and 100 mg/kg quercetin did not suppress the LPS-induced cytokine secretion in serum (Figure 6). In contrast, LPS administration led to the death of all mice from endotoxemia within 4 days and pretreatment with a single dose of quercetin (1, 10, 50, or 100 mg/kg) resulted in a significant protection from lethal septic shock (Figure 7A). Most mice that received acute quercetin treatment were alive at 4 weeks after LPS injection; these findings indicate that quercetin is able to protect mice from endotoxemia when administered in an acute situation. Furthermore, while the LPS-treated mice showed a dramatic increase in their serum levels of TNF-α and IL-1β, pretreatment with quercetin significantly attenuated the serum level of TNF-α at 1 h (Figure 7B) and the serum level of IL-1β at 8 h (Figure 7C). These results show that acute administration of quercetin significantly increases the survival rate of mice at risk of LPS-induced septic death and that these effects are exerted via an anti-inflammatory effect on cytokine production.

## Discussion

Overproduction of TNF-α and IL-1β leads to tissue damage, multiple organ failure, and finally causes lethal sepsis [21]. Therefore, agents attenuating TNF-α and/or IL-1β expression may have potential as treatments for prevention of lethal sepsis [22]. In the present study, we found that treatment of mice with a single dose of quercetin followed one hour later effectively attenuates LPS-induced inflammatory cytokine expression and endotoxemic lethality. This observation is in agreement with those of Tang et al., who reported that quercetin pretreatment for 30 min can prevent endotoxin lethality [20]. On the other hand, we observed that chronic pretreatment of quercetin does not inhibit LPS-induced inflammation. TNF-α and IL-1β are immediately released during the development of systemic inflammatory responses [22]; this leaves a short therapeutic window for treatment. This rapid event explains why we observed that acute administration of quercetin is more efficient and effective than chronic treatment when suppressing the development of inflammation.

In this study, we also investigated the molecular mechanisms by which quercetin down-regulates the LPS-induced inflammatory responses in macrophages. NF-κB plays an essential role in the regulation of inflammatory processes [13]. Both TNF-α and IL-1β are NF-κB target genes and the expressions of these two target genes are remarkably increased in the LPS-stimulated macrophages [23]. We demonstrate that quercetin down-regulates NF-κB activation as well as TNF-α and IL-1β expression in LPS-challenged macrophages. We also observe that quercetin significantly suppresses the phosphorylation of IKK and IκBα as well as the subsequent nuclear translocation of NF-κB subunits p65 and p50. This observation is in agreement with the previous finding that quercetin inhibits IKK activity *in vitro* [24] and suppresses inducible nitric oxide synthase expression by attenuating IKK activity in mouse microglia [25]. Our study clearly shows that quercetin is able to inhibit the NF-κB signaling pathway and this is accompanied by an alleviation of LPS-stimulated TNF-α and IL-1β expressions in macrophages; these events provide a possible mechanism by which quercetin prevents LPS-induced lethality in mice with sepsis.

The Akt pathway has been suggested to activate the NF-κB pathway [26-29]. Choi et al. reported that curcumin, a member of the flavonoids, down-regulates the *mdr1b* gene by attenuating the phosphatidyinositol 3-kinase (PI3K)/Akt/NF-κB pathway [30]. A recent study showed that an Akt inhibitor is able to suppress the plasmin-induced phosphorylation of IκBα in human dendritic cells [31]. Thus, there is accumulating evidence to indicate that Akt may play a role in the regulation of NF-κB activation. In the present study, we found that LPS-induced Akt phosphorylation is decreased by quercetin. This result is in agreement with those of Gulati et al., who reported that quercetin inhibits Akt activation in human breast cancer cells [32]. Furthermore, we demonstrate that quercetin is also able to attenuate the phosphorylation of JNK, which is induced by LPS in macrophages, whereas the phosphorylation levels of ERK and p38 are not changed. Our result is similar to the finding from Zhang et al., who reported that kakkalide, a member of the flavonoid family, is able to down-regulate palmitate-induced TNF-α production via suppression of JNK and NF-κB activation in human umbilical vein endothelial cells [33]. Furthermore, Bennett et al. show that inhibition of JNK with a pharmacological inhibitor blocks LPS-induced TNF-α expression [34]. However, another short-term experiment revealed by Tang et al. has shown that quercetin inhibits ERK, JNK and p38 activation in LPS-challenged RAW264.7 macrophages [20]. Compared with our results, it could suggest that quick changes in the activation of signaling pathways are responsible for the time of quercetin administration at acute phase of inflammation. The short-term phosphorylation changes should probably be regarded as non-genomic effects that could be regulated differently along the agent treating time. Moreover, small interfering RNAs (siRNAs) targeting JNK1 and JNK2 have been shown to decrease free fatty acid-induced IL-1β expression [35]. Taken together, we conclude that the suppression of LPS-induced TNF-α and IL-1β expression by quercetin may also be mediated via the inhibition of JNK activation.

In summary, the present study clearly shows that quercetin markedly reverses LPS-induced TNF-α and IL-1β expressions along with the suppression of NF-κB, Akt, and JNK activation in RAW264.7 macrophages. Moreover, short-term pretreatment of quercetin results in a suppression of TNF-α and IL-1β expressions and this is able to alleviate mortality due to endotoxemic in mice. Therefore, quercetin seems to have therapeutic potential for the protection of systemic inflammatory diseases such as sepsis.
